# Eosinophils Promote Epithelial to Mesenchymal Transition of Bronchial Epithelial Cells

**DOI:** 10.1371/journal.pone.0064281

**Published:** 2013-05-21

**Authors:** Atsushi Yasukawa, Koa Hosoki, Masaaki Toda, Yasushi Miyake, Yuki Matsushima, Takahiro Matsumoto, Daniel Boveda-Ruiz, Paloma Gil-Bernabe, Mizuho Nagao, Mayumi Sugimoto, Yukiko Hiraguchi, Reiko Tokuda, Masahiro Naito, Takehiro Takagi, Corina N. D'Alessandro-Gabazza, Shigeru Suga, Tetsu Kobayashi, Takao Fujisawa, Osamu Taguchi, Esteban C. Gabazza

**Affiliations:** 1 Department of Immunology, Mie University Graduate School of Medicine, Tsu, Mie, Japan; 2 Department of Pulmonary and Critical Care Medicine, Mie University Graduate School of Medicine, Tsu, Mie, Japan; 3 Institute for Clinical Research, Mie National Hospital, Tsu City, Mie, Japan; National Jewish Health, United States of America

## Abstract

Eosinophilic inflammation and remodeling of the airways including subepithelial fibrosis and myofibroblast hyperplasia are characteristic pathological findings of bronchial asthma. Epithelial to mesenchymal transition (EMT) plays a critical role in airway remodelling. In this study, we hypothesized that infiltrating eosinophils promote airway remodelling in bronchial asthma. To demonstrate this hypothesis we evaluated the effect of eosinophils on EMT by *in vitro* and *in vivo* studies. EMT was assessed in mice that received intra-tracheal instillation of mouse bone marrow derived eosinophils and in human bronchial epithelial cells co-cultured with eosinophils freshly purified from healthy individuals or with eosinophilic leukemia cell lines. Intra-tracheal instillation of eosinophils was associated with enhanced bronchial inflammation and fibrosis and increased lung concentration of growth factors. Mice instilled with eosinophils pre-treated with transforming growth factor(TGF)-β1 siRNA had decreased bronchial wall fibrosis compared to controls. EMT was induced in bronchial epithelial cells co-cultured with human eosinophils and it was associated with increased expression of TGF-β1 and Smad3 phosphorylation in the bronchial epithelial cells. Treatment with anti-TGF-β1 antibody blocked EMT in bronchial epithelial cells. Eosinophils induced EMT in bronchial epithelial cells, suggesting their contribution to the pathogenesis of airway remodelling.

## Introduction

Bronchial asthma is a chronic inflammatory disorder of the airways with a high worldwide prevalence [1]. Bronchial eosinophilic inflammation is a cardinal pathological feature of acute asthma [2], and airway remodeling, that is characterized by subepithelial fibrosis, myofibroblast hyperplasia, thickening of the lamina reticularis and increased smooth muscle mass, is a common consequence of chronic asthma [3]. The number of myofibroblasts increases in the proximity of the smooth muscle layer and the lamina reticularis in asthmatic patients [4]. They are rich source of collagens types I, III, and V, fibronectin and tenascin that accumulate in the airway walls causing thickening of the lamina reticularis [5], [6]. Myofibroblasts may derive from resident fibroblasts and/or blood-circulating fibrocytes [7] and and/or epithelial cells by epithelial to mesenchymal transition (EMT). EMT is currently recognized as an important mechanism for the increased number of myofibroblasts in cancer and fibrotic diseases [8].

Transforming growth factor (TGF)-β1 is involved in the pathogenesis of airway remodeling [9]; increased level of TGF-β1 has been reported in bronchoalveolar lavage fluid (BALF) and in bronchial biopsy tissues from asthmatic patients [10]. The source of TGF-β1 in the lungs is not completely clear; some studies have shown that eosinophils, epithelial cells and fibroblasts can secrete TGF-β1 at sites of injury [11]. TGF-β1 can promote airway remodelling by inducing EMT in epithelial cells [12]. In patients with idiopathic pulmonary fibrosis, TGF-β1 induces EMT in alveolar epithelial cells and it is believed to be a major contributor to enhanced fibrosis [13]. However, little is known about EMT in asthma and whether the increased TGF-β1 expression is involved in its pathogenesis. A recent study has shown that EMT may cause airway remodelling in asthma [14]. Hackett et al suggested that the number of airway epithelial cells undergoing EMT is enhanced and that the process of epithelial repairs is dysregulated in bronchial asthma [14]. Johnson et al reported that house dust mite exposure induces EMT in large airways in mice [15]. These findings suggest that TGF-β1 released by eosinophils may induce EMT leading to airway remodelling.

In the present study, we hypothesized that infiltrating eosinophils in the lungs induce EMT in bronchial epithelial cells through the TGF-β1 signalling pathway, and thereby contribute to the mechanism of airway remodelling in chronic bronchial asthma.

## Results

### Intra-tracheal instillation of eosinophils induced peribronchial fibrosis

**Figure 1:**
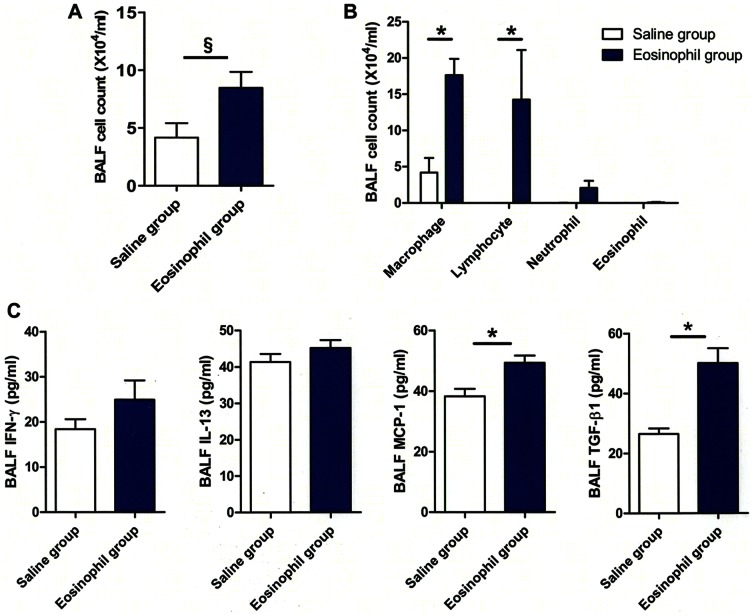
Inflammatory cells and elevation of cytokines in eosinophil-treated mice. (A) The total cell count in BALF was significantly increased in the group receiving instillation of eosinophils compared to control mice (each group, n = 4). (B) Differential cell count showed significantly increased number of macrophages and lymphocytes in eosinophil-instilled mice compared to controls. (C) Mice intratracheally instilled with eosinophils had significantly elevated levels of MCP-1/CCL2 and TGF-β1 in BALF compared to controls. IFN-γ and IL-13 showed no significant change. Data are expressed as means ± SEM. *P<0.05; ^§^P = 0.05.

First, experiments were conducted to test whether the presence of increased number of eosinophils in the airways induces pathological changes in the lungs *in vivo*. Intra-tracheal instillation of bone marrow-derived eosinophils led to increased number of total cell count, macrophages and lymphocytes in bronchoalveolar lavage fluid (BALF) on day 9 as compared with mice receiving saline instillation (**Figure 1A, B**). As shown in **Figure 1C** intra-tracheal instillation of eosinophils was associated with increased IFN-γ and IL-13 in BALF as compared to the saline group but the differences did not reach statistical significance. TGF-β1 and MCP-1 in BALF were significantly elevated in mice that received intra-tracheal instillation of eosinophils compared with the saline group.

**Figure 2:**
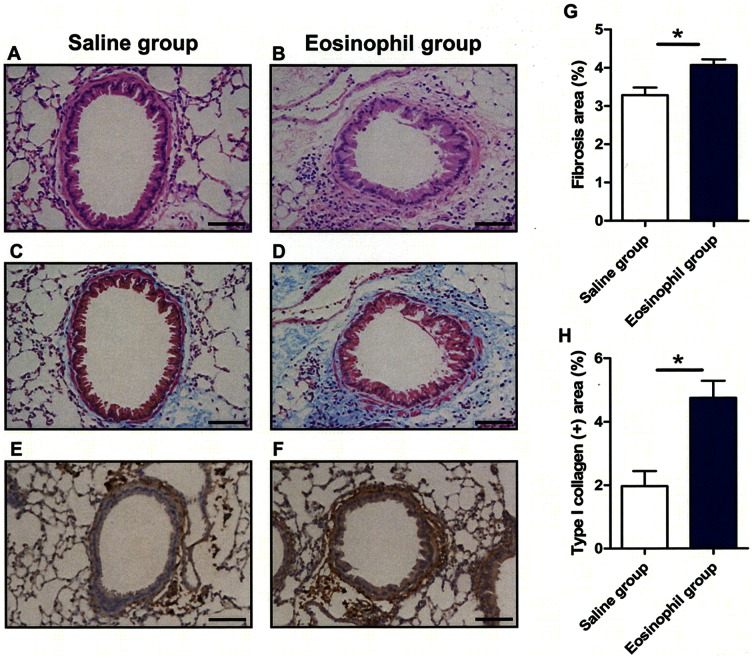
Inflammation and fibrosis of the airway walls induced by eosinophils instillation *in vivo*. (A–F) Representative lung sections from control (n = 3; A, C, E) and eosinophil-instilled mice (n = 4; B, D, F). Specimens were stained with Hematoxylin-Eosin (A, B), Masson trichrome (C, D) and immunostained for type I collagen (E, F) and examined by light microscopy. Mice receiving intra-tracheal eosinophils had increased infiltration of inflammatory cells around the bronchi (B), peribronchial fibrosis (D) and deposition of type I collagen (F) in lung tissue compared to controls (A, C and E). (G, H) Collagen deposition (G) and immunoreactive areas for type I collagen (H) were quantified using WinROOF software. Lung tissue of eosinophil-instilled mice showed significant deposition of collagen as compared to control. The data are expressed as the mean percentage of the total lung field area ± S.E.M. The scale bars indicate 50 µm. Data are expressed as means ± SEM. *P<0.05.

In a separate experiment, the lungs were pathologically examined after 21 days of eosinophil instillation (**Figure 2A, B, C, D**). Enhanced type I collagen deposition was detected in eosinophil-instilled mice (**Figure 2E, F**) and the areas of fibrosis and collagen I deposition were significantly increased in eosinophil-instilled mice compared with the saline group (**Figure 2G, H**).

### EMT is induced by eosinophils *in vivo*

**Figure 3:**
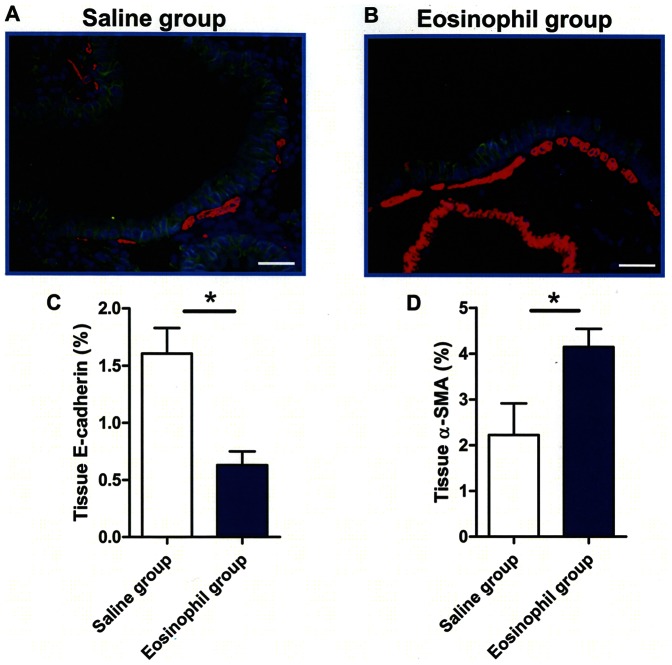
Induction of epithelial–mesenchymal transition in the airways by eosinophils *in vivo*. (A, B) Representative immunofluorescence staining of E-cadherin (green), α-SMA (red) and nuclei (blue) in the airways of mice instilled with saline (A) or eosinophils (B). (C, D) Quantification by densitometry disclosed low expression of E-cadherin (C) and increased expression of α-SMA (D) in mice after eosinophil instillation (n = 4) compared to control (n = 3). The data are expressed as the mean percentage of the total lung field area ± SEM. The scale bars indicate 50 µm. Data are expressed as means ± SEM. *P<0.05.

EMT is defined by changes in gene or protein expressions; during EMT epithelial markers (e.g. E-cadherin) decrease while mesenchymal markers (e.g. α-SMA, vimentin) increase. The bronchial walls of mice receiving instillation of eosinophils showed significantly less expression of E-cadherin but more expression of α-SMA on day 21 than those of saline-treated mice (**Figure 3A, B, C, D**).

### Diffuse distribution and early lung inflammation after eosinophil instillation

Eosinophils were observed in the bronchial walls and alveolar interstitial spaces of both lungs 3 h after intra-tracheal instillation (**Figure S1 A**). Evaluation of the time course of differential cells after intra-tracheal instillation of eosinophils showed a relative increase of lung eosinophils at 6 h and 24 h after instillation with almost complete disappearance of them at 72 h of instillation. There was a relative increase in the number of lymphocytes after 72 h of eosinophil instillation. Histological examination showed enhanced mononuclear infiltration in peribronchial areas at 24 h and 72 h of eosinophil instillation (**Figure S1 B, C**). The concentration of TGF-β1 in BALF also tended to increase 24 h and 72 h after eosinophil instillation compared to control mice (**Figure S1 D**).

### Human eosinophils induced morphological changes in bronchial cells

**Figure 4:**
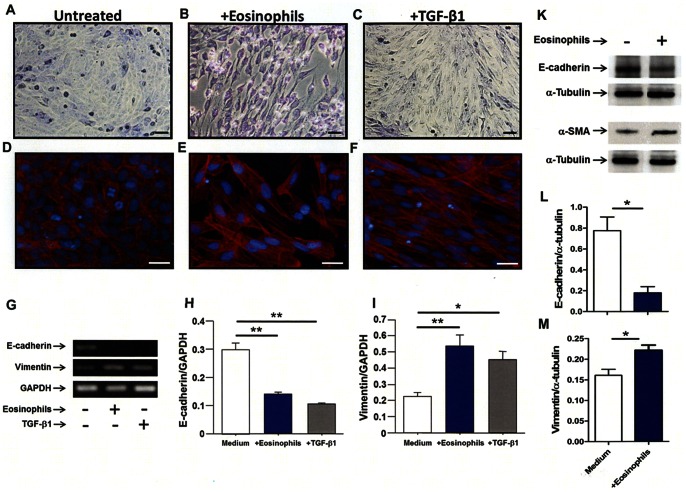
Human eosinophils induced EMT morphological changes in BEAS-2B cells. (A, D) control, (B, E) BEAS-2B cells co-cultured with eosinophils. (C, F) BEAS-2B cells cultured in the presence of TGF-β1. BEAS-2B cells were stained with Diff-Quick after washing out eosinophils (A, B, C). (D, E, F) Immunofluorescence staining was performed with phalloidin (red) and DAPI for nuclei staining (blue) after washing out eosinophils. The scale bars indicate 50 µm. (G, H, I) RNA expression of E-cadherin and vimentin in BEAS-2B cells co-cultured with or without eosinophils and in the presence of TGF-β1 as assessed by RT-PCR and densitometry analysis. (K, L, M) Protein expression of E-cadherin and αSMA in BEAS-2B cells co-cultured with or without eosinophils as assessed by Western blotting and densitometry analysis. Data are expressed as means ± SEM. **P<0.01; *P<0.05.

The *in vitro* experiments demonstrated that human eosinophils induce EMT in the human bronchial epithelial cell line BEAS-2B cells. As shown in **Figure 4 (A, B, C)** BEAS-2B cells co-cultured with human eosinophils or in the presence of TGF-β1 exhibited fibroblast-like morphology consistent with EMT, while BEAS-2B cells cultured in medium alone conserved the typical epithelial cobblestone pattern. Moreover, we confirmed filamentous actin (F-actin) forming long stress fibers in BEAS-2B cells co-cultured with human eosinophils or cultured in the presence of TGF-β1 using fluorescence microscope (**Figure 4D, E, F**). PCR and Western blot analyses also confirmed the development of EMT by showing significantly decreased expression of E-cadherin and increased expression of vimentin in cells co-cultured with human eosinophils (Figure 4G, H, I); the RNA expression of both EMT markers was also similarly changed in the presence of TGF-β1 (Figure 4G, H, I).

### EMT induced by eosinophils is TGF-β1 dependent

**Figure 5:**
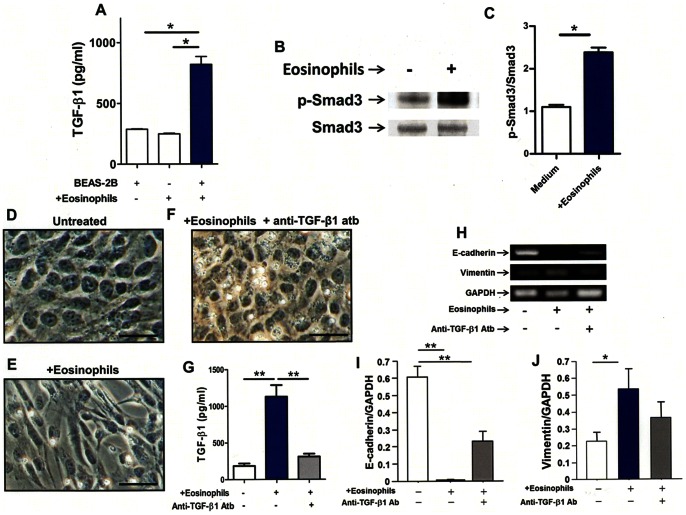
Eosinophils induced secretion of TGF-β1 from BEAS-2B cells. (A) TGF-β1 levels in the supernatant from each cell condition. (B) Representative Western blotting of Smad3 and p-Smad3. (C) The ratio of p-Smad3 to Smad3 analyzed by densitometry. (D, E, F) Morphological changes in each cell condition. (G, H, I, J) Effect of anti-TGF-β1 antibody on the protein level of TGF-β1 and RNA expression of E-cadherin and vimentin during each cell condition. BEAS-2B cells were stained with DIFF-QUICK. The scale bars indicate 50 µm. Data are expressed as means ± SEM. *P<0.05.

We found that co-culture of human eosinophils with BEAS-2B cells significantly enhances the production of TGF-β1 compared with BEAS-2B cells or eosinophils alone (**Figure 5A**). Western blotting revealed that the ratio of p-Smad3 to Smad3 in BEAS-2B cells was significantly enhanced by eosinophils (**Figure 5B, C**). Next, we examined whether the EMT in our co-culture system is TGF-β1 dependent. The characteristic morphological changes of EMT in BEAS-2B cells co-cultured with eosinophils were abolished in the presence anti-TGF-β1 mAtb (**Figure 5D, E, F**). Anti-TGF-β1 mAtb also significantly inhibited the increase of TGF-β1 production and the decreased expression of E-cadherin, and tended to inhibit the increase of vimentin expression (**Figure 5G, I, J**).

**Figure 6:**
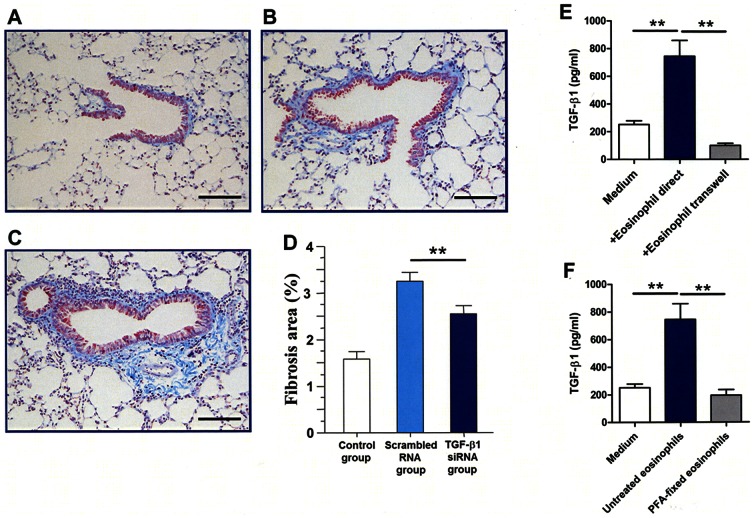
Relevant role of TGF-β1 in airway remodeling and the need of direct contact of eosinophils for TGF-β1 production. (A, B, C) Mice receiving instillation of eosinophils pre-treated with TGF-β1 siRNA have less peribronchial fibrosis than those receiving eosinophils pre-treated with scrambled RNA as assessed by Masson staining. (D) Quantification of Masson positive areas disclosed significant difference. (E, F) TGFβ1 levels in the supernatant in Boyden chamber assay and using 2% paraformaldehyde-fixed eosinophils. Culture supernatants were used to measure TGF-β1 by ELISA. Data are expressed as means ± SEM. **P<0.05. The scale bars indicate 100 µm. Data are expressed as means ± SEM. **P<0.05.

To further demonstrate the biological relevance of TGF-β1 in the mechanism of airway remodeling, mice were instilled with bone marrow-derived eosinophils pre-treated with TGF-β1 siRNA or scrambled RNA and the development of airway remodeling was compared. Mice that were instilled eosinophils pre-treated with TGF-β1 siRNA had significantly less airway remodeling than mice instilled with eosinophils pre-treated with scrambled RNA (**Figure 6A, B, C, D**).

### EMT requires cell-to-cell contact and eosinophil activation

To test whether cell-to-cell contact is necessary for EMT induction, we used a trans-well system to inhibit direct cell-to-cell contact between BEAS-2B cells and eosinopohils. Eosinophils could not induce EMT in BEAS-2B cells (data not shown) or TGF-β1 production in the absence of cell contact (**Figure 6E**). Moreover, we fixed eosinophils by paraformaldehyde before co-culture and tested whether fixed eosinophils induces EMT in BEAS-2B cells. Neither EMT of BEAS-2B cells (data not shown) nor increase in TGF-β1 production was detected in cell supernatant using fixed eosinophils (**Figure 6F**).

### EoL-1 cells induce EMT in BEAS-2B cells

**Figure 7:**
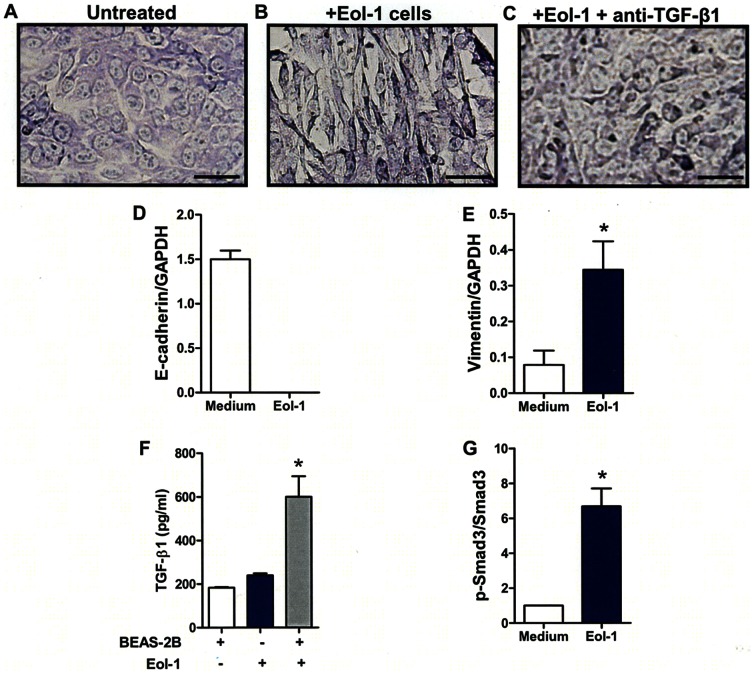
Eol-1 cell lines induce EMT in BEAS-2B cells by increasing TGF-β1 expression. (A, B) Control and BEAS-2B cells co-cultured with EoL-1 cells. (C) BEAS-2B cells co-cultured with EoL-1 cells in the presence of anti-TGF-β1 neutralizing antibody. BEAS-2B cells lysates were subjected to RT-PCR analysis. The expression level of E-cadherin (D) and vimentin (E) mRNA was measured by RT-PCR and normalized to that of GAPDH mRNA. TGF-β1 (F) levels in the supernatant from each culture condition, and the ratio of p-Smad3 to Smad3 (G) in BEAS-2B cells as analyzed by densitometry. The scale bars indicate 50 µm. Data are expressed as means ± SEM. *P<0.05.

Like primary human eosinophils, the human eosinophilic leukemia cell line, EoL-1 cells, also induced EMT in BEAS-2B cells (**Figure 7A, B**). EMT changes in BEAS-2B cells induced by Eol-1 were also inhibited in the presence of anti-TGF-β1 mAb (**Figure 7C**). Changes in the mRNA expression of E-cadherin and vimentin observed in BEAS-2B cells co-cultured with Eol-1 were similar to those observed when the epithelial cell line was cultured with primary human eosinophils (**Figure 7D, E**). Further, the protein level of TGF-β1 was significantly increased in the co-culture supernatant (**Figure 7F**) and the p-Smad3 to Smad3 ratio was significantly high in BEAS-2B cells cultured with Eol-1 cells (**Figure 7G**). The need of cell-to-cell contact for induction of EMT in BEAS-2B by EoL-1 cells was also appraised using a trans-well system. Eol-1 cells were unable to induce EMT in BEAS-2B cells or to increase TGF-β1 production in the absence of contact (**Figure S2 A, B, C**). Neither EMT of BEAS-2B cells nor increase of TGF-β1 production was observed when fixed Eol-1 cells were used in the experiments (**Figure S2 D, E, F**).

### JNK pathway mediates TGF-β1expresion during co-culture

**Figure 8:**
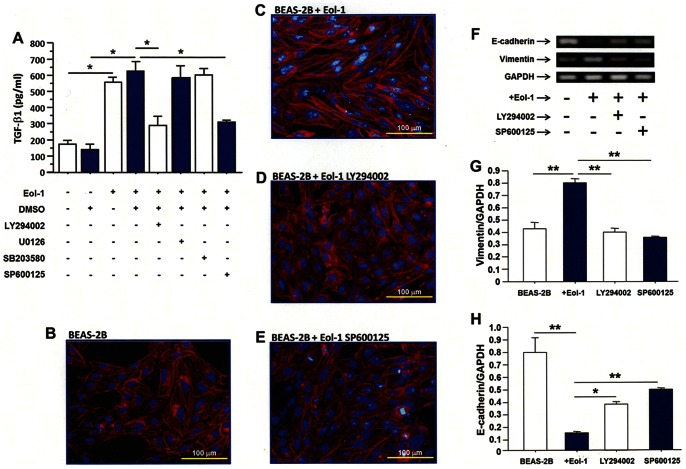
Eol-1 cells induce EMT in BEAS-2B cells via the JNK/Smad3 pathway. (A) The concentration of TGF-β1 in the supernatant of BEAS-2B in the presence of PI3 kinase (LY294002), JNK (SP600125), MEK1/2 (MUO126) or p38 kinase (SB203580) inhibitor. Inhibitors of both PI3 and JNK kinases suppress the morphological features (B, C, D, E) and molecular markers (F, G, H) of EMT. Data are expressed as means ± SEM. *P<0.05; **P<0.01.

The concentration of TGF-β1 in the supernatant of BEAS-2B cells co-cultured with Eol-1 cells was significantly inhibited in the presence of PI3 kinase (LY294002) or JNK (SP600125) inhibitors but not in the present of MEK1/2 (MUO126) or p38 kinase (SB203580) inhibitors (**Figure 8A**). Morphological features (**Figure 8B, C, D, E**) and the RNA expression of molecular markers (**Figure 8F, G, H**) of EMT were also significantly blocked by both PI3 and JNK inhibitors.

## Discussion

Eosinophils are well recognized to play a critical role in the pathogenesis of asthma during the initiation and acute exacerbation of the disease, but their contribution to airway remodelling and fibrosis has not been so far demonstrated [16], [17]. This study provides the first evidence that eosinophils induce fibrosis and EMT of bronchial epithelial cells in the airways.

### Eosinophils and recruitment of inflammatory cells into the airways

In the current study, we found a significant increase in the total cell count, in the number of macrophages and lymphocytes and a significant increase in the lung concentration of the chemokine MCP-1/CCL in mice on day 9 after receiving intratracheal instillation of eosinophils compared to control mice. The explanation for this finding is unclear. Jacobson et al previously reported that lung infiltration and the secretion of chemokines by eosinophils are required for the recruitment of effectors T cells during allergic pulmonary inflammation [18]. Based on this previous observation and knowing that MCP-1/CCL2 is a potent chemoattractant for macrophages and lymphocytes, it is conceivable that MCP-1/CCL2 secreted from the lungs induced the local recruitment of leukocytes after intratracheal instillation of eosinophils [19]. Interestingly, lymphocytes started to increase in the lungs after 24 h of eosinophil instillation, at the time when eosinophils almost disappeared. Shi et al previously reported that eosinophils instilled into the trachea migrate to draining peritracheal lymph nodes within 24 h in mice [20]; and this finding may explain why eosinophils could not be detected in our mouse *in vivo* model 9 days after intratracheal instillation.

### Inflammatory cells and airway remodeling

Airway remodelling results from chronic inflammation of the airways that is characterized by subepithelial fibrosis, myofibroblast hyperplasia, goblet cell hyperplasia, thickening of the lamina reticularis and smooth muscle hypertrophy and hyperplasia [3], [21]; airway remodelling is frequently associated with severe chronic asthma and when it is present the disease poorly responds to conventional anti-inflammatory therapies [22]. EMT of airway epithelial cells has been shown to be an important contributor to the process of airway remodelling because it increases the number of mesenchymal cells migrating to the sub-epithelial connective tissue where they enhance the production of extracellular matrix proteins causing bronchial wall fibrosis [23]. Inflammatory cells have been reported to promote the development of EMT. Monocytes/macrophages can induce EMT of renal epithelial cells during unilateral ureteral obstruction, T cells can induce EMT of renal epithelial cells and liver biliary cells during allograft rejection and eosinophils have been involved in EMT of oesophageal mucosal epithelial cells [24]–[27]. Growth factors (e.g., TGF-β1) and adhesion molecules (e.g., ICAM-1) have been reported to be involved in EMT induced by inflammatory cells [22]. In the present study, mice that received intratracheal instillation of eosinophils showed significantly low immunoreactivity for E-cadherin, high fibrosis score with increased staining of α-SMA and collagen in the airway walls, suggesting that eosinophils induce EMT in airway epithelial cells and contribute to airway remodelling.

### Eosinophils, TGF-β1 and EMT

TGF-β1 is the major inducer of EMT and therefore the most active player in the process of airway remodeling [19], [22]. Several clinical studies have shown that the expression of TGFβ-1 is enhanced in patients with bronchial asthma and that it is correlated with the number of infiltrating eosinophils and the degree of airway remodeling [22], [28], [29]. However, to date evidence showing that eosinophils are directly involved in the induction of EMT in bronchial epithelial cells is lacking. To address this question, in the present study we cultured human bronchial epithelial cells with eosinophils freshly isolated from human volunteers and studied changes in EMT markers. After co-culture with human eosinophils, cobblestone-like bronchial epithelial changed to fibroblast-like cells, expressed more mesenchymal (vimentin) than epithelial cell markers (E-cadherin) on their cell surface and secreted significant amount of TGF-β1 compared to cells cultured alone, suggesting that eosinophils induce EMT and increase the secretion of TGF-β1 in bronchial epithelial cells. The significant and strong inhibition of the morphological changes of EMT in the presence of anti-TGF-β1 antibody during co-culture of bronchial epithelail cells with primary eosinophils or Eol-1, and the decreased airway remodeling in mice instilled with eosinophils pre-treated with TGF-β1 siRNA, provide evidence that TGF-β1 mediates the EMT induced by eosinophils in bronchial epithelial cells.

### TGF-β1/smad3 pathway and EMT

Previous studies have shown that TGF-β1 induces EMT by activating the TGF-β1/smad3 signalling pathway [22], [30]. TGF-β1 activates cells by binding to the type II TGF-β receptor that forms a dimer with Type I TGF-β1 receptor [31]. Tretramerization of the two different receptor serine/threonine kinases activates the receptor signalling leading to phosphorylation of cytoplasmic signal transducers Smad2 and Smad3 [30]. In the presence study, bronchial epithelial cells cultured with eosinophils isolated from humans or with Eol-1 cells showed enhanced activation of Smad3, suggesting that the TGF-β1/Smad3 signalling is also involved in EMT induction by eosinophils. The c-Jun N terminal kinase (JNK) has been also implicated in TGFβ1-induced EMT [19], [32]. In agreement with this, significant inhibition of TGF-β1 secretion, and significant blockade of morphological features and expression of molecular markers of EMT in bronchial epithelial cells by the addition of a specific inhibitor of the JNK pathway during co-culture of bronchial cells and eosinophils were observed in our present co-culture model, suggesting the role of JNK pathway in EMT induced by eosinophils. Overall, these observations suggest that interaction of eosinophils with airway epithelial cells induces secretion of TGF-β1 which activates the JNK/Smad3 pathway causing EMT of the airway epithelial cells.

### Eosinophil surface contact and EMT

Eosinophils can express several inflammatory mediators including cytokines and growth factors [21]. In this study we showed that the cell supernatant of primary eosinophils or Eol-1 cells co-cultured with bronchial epithelial cells contained high concentration of TGF-β1. To demonstrate whether a direct cell contact is required for the induction of soluble factors and EMT by eosinophils, the cells were cultured using a trans-well system and found that neither increase in the level of supernatant TGF-β1 nor induction of EMT occurred. These observations suggest the need of eosinophil contact to induce EMT of bronchial epithelial cells.

### Conclusion

The results of this study show that cell-to-cell contact of eosinophils infiltrating the lungs promotes increases in the expression of TGF-β1 leading to activation of the JNK/Smad3 pathway and induction of EMT in airway epithelial cells.

## Materials and Methods

### Ethics Statement

The Mie University's Committee on Animal Investigation approved the experimental protocol (No 24-1; 2012.6.19). The study was approved by the Institutional Ethic Board for Clinical Investigation (No 1004; 2008.1.9), written informed consent was obtained from all healthy volunteers before blood sampling and the study was performed following the principles of Helsinki Declaration.

### Reagent

Stromal cell factor (SCF), Flt3-ligand and IL-5 were obtained from PeproTech (Princeton, NJ). L-glutamine, penicillin/streptomycin, donkey anti-mouse IgG-Alexa Fluor 488, chicken anti-rabbit IgG-Alexa Fluor 594, Texas Red-X phalloidin, Laemmli sample buffer and Trizol Reagent were purchased from Invitrogen (Carlsbad, CA). RPMI-1640 and bovine serum albumin (BSA) were from Sigma (St Louis, MO). Fetal bovine serum (FBS) was obtained from BioWhittaker (Walkersville, MD, USA). Rabbit anti–human α-SMA antibody, goat anti-human E-cadherin antibody [HECD-1] and rabbit anti-human α-SMA polyclonal antibody were purchased from Abcam (Cambridge, MA). Mouse anti–human E-cadherin antibody was obtained from BD Biosciences (Mississauga, ON, Canada). Rabbit anti-collagen type I antibody was from ROCLAND (Ontario, Canada). Anti-TGF-β1 monoclonal antibody (mAb) (1D11) was obtained from R&D Systems (Minneapolis, MN). Anti-human Smad3 antibody and rabbit anti-human phospholyration-Smad3 (p-Smad-3) polyclonal antibody (Ser423/425) were from Cell Signaling Technology (Beverly, MA). Mouse anti-human α- tubulin monoclonal antibody (mAb) (B-7) and RIPA lysis buffer was purchased from Santa Cruz Biotechnology (Santa Cruz, CA). Horseradish peroxidase-conjugated goat anti-rabbit IgG and horseradish peroxidase-conjugated goat anti-mouse IgG were from BIO-RAD (Hercules, CA). Anti-CD16 and anti-CD14 bound micromagnetic beads were obtained from Miltenyi Biotec (Auburn, CA). The phosphatidylinositol 3 (PI3) kinase inhibitor (LY294002), mitogen-activated protein kinase kinase (MEK1/2) inhibitor (MUO126), p38 kinase inhibitor (SB203580) or c-Jun NH2-terminal kinase (JNK) inhibitor (SP600125) were purchased from Calbiochem (San Diego, CA).

### Animals

Nine-week-old, male C57/BL6 mice that weighed 23–24 g were purchased from Nihon SLC and housed in the animal facility of Mie University. Mice were maintained on a constant 12-h light/12-h dark cycle in a temperature- and humidity-controlled room and were given *ad libitum* access to food and water.

### Intra-tracheal instillation of bone marrow-derived eosinophils

Differentiated eosinophils from C57/Bl6 mice bone marrow progenitors were prepared using modification of a method reported elsewhere [33]. Briefly, bone marrow cells were collected from the femurs and tibiae of wild-type BALB/c mouse, and then red blood cells were lysed using ACK (Ammonium chloride, Kalium hydrogen carbonate, ethylenediaminetetraacetic acid) buffer. After washing, bone marrow cells were cultured in medium containing RPMI 1640 with 10% FBS, 100 IU/ml penicillin and 10 µg/ml streptomycin, and 50 µM 2-mercaptoethanol (Sigma-Aldrich) supplemented with 100 ng/ml SCF and 100 ng/ml FLT3-ligand for 4 days. After that, medium containing SCF and FLT3-ligand was replaced with that containing 10 ng/ml recombinant mouse IL-5. After incubation with IL-5 for 3 days, the cells were harvested and confirmed to be differentiated eosinophils by flow cytometry. The purity of eosinophils was more than 90%. Under pentobarbital anesthesia, mice received intra-tracheally either bone marrow-derived eosinophils (2×10^6^/cells in 100 µl saline) or sterile saline (100 µl) on day 0, 7 and 14; for intra-tracheal instillation, we used high number of eosinophils because we have previously detected between 1 to 2×10^6^ eosinophils in mouse models of bronchial asthma [34]. Mice were sacrificed and samples were taken either on day 9 or 21 for subsequent analysis.

### Sampling of bronchoalveolar lavage fluid (BALF) and lung specimens

Mice were subjected to euthanasia by intraperitoneal pentobarbital overdose and samples for biochemical and histological examinations were taken. BALF samples were drawn as previously described [35]. The total number of cells in BALF was measured using a nucleocounter from ChemoMetec (Allerod, Denmark). The BALF supernatant was separated by centrifugation and stored at −80°C until use for biochemical analysis. For differential cell count, BALF was centrifuged using a cytospin and the cells were stained with May-Grunwald-Giemsa (Merck, Darmstadt, Germany). Mice were thoracotomized and the pulmonary circulation flushed with saline and then the lungs were excised. The lung was perfused with 10% neutral buffered formalin, fixed in formalin for 24 h and then embedded in paraffin.

### Time course of differential cell count after intra-tracheal instillation of eosinophils

Mice received intra-tracheal instillation of eosinophils (2×10^6^ cells) and then sacrificed by anesthesia overdose on days 0 h (n = 3), 6 h (n = 3), 24 h (n = 3) and 72 h (n = 3). BALF sampling and processing was performed as described above.

### Histological examination

Five-micrometer thick sections of lung specimens were prepared and stained with Hematoxylin-Eosin or Masson trichrome and then examined under light microscopy (Olympus BX50 microscope; Tokyo, Japan). Type I collagen was also immunostained. Briefly, deparaffinized tissue sections were subjected to hydrated autoclaving for antigen retrieval. The tissue sections were immersed in 1% hydrogen peroxide for 30 min to block endogenous peroxidase activity and nonspecific binding was blocked with 10% goat serum in phosphate-buffered saline (PBS) for 1 h. After washing, slides were exposed to rabbit anti-collagen type I antibody in blocking buffer for 1 h at 37°C and 5% CO_2_. Secondary antibody (anti-mouse or anti-rabbit antibody) labelled with biotin was subsequently applied and incubated for 2 h at room temperature, followed by treatment with the avidin–biotin complex peroxidase (Vectastain ABC Kit, Vector Laboratories, Burlingame, CA). Development was then performed using a 3, 3′-diaminobenzidine tetrahydrochloride solution and then counterstained with hematoxylin.

For immunofluorescence, deparaffinized tissue sections were subjected to hydrated autoclaving for antigen retrieval. After washing with Tris-buffered saline, slides were exposed to mouse anti–human E-cadherin antibody (1∶200) overnight at 4°C and subsequently incubated with donkey anti-mouse IgG-Alexa Fluor 488 (1∶200) for 4 h at room temperature after washing. Staining of α-SMA was done using rabbit anti–human α-SMA antibody (1∶200) and then chicken anti-rabbit IgG-Alexa Fluor 594 (1∶200). After washing, the sections were counterstained with 4,6-diamidino-2-phenylindole (DAPI) and mounted using a fluorescence mounting medium.

A blinded researcher randomly took the same number of photos of lung fields including bronchial walls from each mouse in all groups using an Olympus BX50 microscope or a fluorescence microscope (IX71 Olympus) combined with an Olympus DP70 digital camera (Tokyo, Japan). The grade of bronchial wall fibrosis was determined by measuring the areas that positively stained for Masson trichrome or for type I collagen in relation to the total area of each lung field taken as 100% using the WinROOF image processing software (Mitani Corp., Fukui, Japan) for Windows. The expression of E-cadherin and α-SMA was quantified by measuring the positively stained areas for each marker in photos of bronchi taken blindly and at random as described above [36].

### Lung distribution of eosinophils after intra-tracheal instillation

Bone marrow-derived eosinophils (2×10^6^ cells) were prepared as described above, labeled with carboxyfluorescein succinimidyl ester (CFSE) and intratracheally instilled into female C57BL/7 mice (n = 3); mice (n = 3) instilled with CFSE alone was used as controls. After 3 h of instillation, both groups of mice were sacrificed by anesthesia overdose, the lungs excised and then fixed in formalin as decribed above. After deparaffinization, the tissue sections were counterstained with DAPI and observed under fluorescent microscope.

### Intra-tracheal instillation of eosinphils pre-treated with TGF-β1 siRNA

Bone marrow-derived eosinophils (2×10^6^ cells) were prepared as described above, cultured in the presence of mouse TGF-β1 siRNA (n = 3) or scrambled RNA (n = 3) for 6 h at 37°C before intra-tracheally instilling into female C57BL/7 mice. Mice instilled with saline were used as controls. The sequence of TGF-β1 siRNA and scrambled RNA were previously described [37]. Mice were sacrificed on the 6^th^ day after eosinophil instillation by anesthesia overdose, lung excised and prepared for H&E and Masson staining as described above.

### Cell lines

BEAS-2B is an adenovirus 12-SV40 virus hybrid (Ad12SV40) transformed human epithelial cell line, which was isolated from normal human bronchial epithelium obtained from the autopsy of a noncancerous individual. It was obtained from the Riken Cell Bank (Tsukuba, Japan), grown in DMEM supplemented with 10% (v/v) heat-inactivated FBS, 0.03% (w/v) L-glutamine, 100 IU/ml penicillin and 100 µg/ml streptomycin. Before each experiment, BEAS-2B cells were cultured in 6-well plate up to confluence. EoL-l cells were obtained from the Riken Cell Bank, maintained in suspension culture at 37°C and 5% CO_2_ in humidified atmosphere using RPMI-1640 medium supplemented with 10% (v/v) heat-inactivated FBS, 0.03% (w/v) L-glutamine, 100 IU/ml penicillin and 100 µg/ml streptomycin. For differentiation, EoL-1 cells were diluted to 5×10^5^ cells/ml and 0.5 mM sodium n-butyrate (BA) was added. When EoL-1 cells were incubated with 0.5 mM BA for 5 days, the percentage of cells that stained positive with the EoProbe Eosinophil Staining Kit (BioFX) increased from 2.6+0.3 to 78.5+4.9%.

### Preparation of human eosinophils

Eosinophils were purified from healthy volunteers after obtaining informed consent by negative selection using anti-CD16 and anti-CD14 bound micromagnetic beads as previously described [38]. The purity of eosinophils was more than 97%.

### Co-culture experiment and morphological analysis

BEAS-2B cells were cultured in 6- or 12-well plate until 60–70% cell confluence, then serum-starved for 24 h. Human eosinophils or BA-differentiated EoL-1 were added to the culture (1×10^6^ cells for 12-well plate, 2×10^6^ cells for 6-well plate) and incubated for further 48 h. In some experiments, co-culture of BEAS-2B cells and eosinophils or Eol-1 were performed in the presence of mouse anti-TGF-β1 mAb (1D11; 30 µg/ml) using a Boyden microchemotaxis chamber (BD-Falcon, Heidelberg, Germany) in which a 0.4-µm-pore-size polyethylene terephthalate (PET) membrane separated the upper and lower chambers. In part of the experiments, Eol-1 or eosinophils were fixed with 2% paraformaldehyde for 15 min and used for co-culture experiment instead of live cells. After co-culture, human eosinophils or Eol-1 cells were removed from adherent BEAS-2B cells by gentle pipetting. BEAS-2B cells were stained with Diff-Quick solutions and photographed for morphological analysis. Furthermore, we stained nuclei and F-actin cytoskeleton using immunofluorescence. Briefly, BEAS-2B was fixed with 4% paraformaldehyde for 10 min at room temperature. After washing, BEAS-2B cells were stained with Texas Red-X phalloidin (1∶50) for 1 h at room temperature; DAPI was used to stain nuclei for 5 min at room temperature. Fluorescent images were captured using a fluorescence microscope (IX71 Olympus) after appropriate washing.

### Western blot

For Western blot analysis, cells were lysed in RIPA (radioimmunoprecipitation) lysis buffer (sc-24948). Cell debris was removed by centrifugation at 15000×g for 15 min, and supernatant was boiled in Laemmli sample buffer for 5 min at 70°C. An equal amount of proteins was subjected to sodium dodecyl sulphate- 10% polyacrylamide gel electrophoresis before blotting onto a PVDF (polyvinylidine fluoride) membrane (Amersham Pharmacia Biotech). The membrane was blocked with 5% bovine serum albumin (BSA) in TBS buffer containing 0.1% Tween-20, 25 mM Tris-HCl, 0.15 M NaCl, pH 7.6 at 4°C overnight and probed with primary goat anti-human E-cadherin antibody [HECD-1], rabbit anti-human α-SMA polyclonal antibody, anti-human Smad3 antibody, rabbit anti-human phospholylation-Smad3 (p-Smad-3) polyclonal antibody (Ser423/425) or mouse anti-human α-tubulin mAb (B-7) for 2 h at room temperature. After washing, the membrane was incubated with secondary goat anti-rabbit or anti-mouse antibody coupled to horseradish peroxidase for 2 h at room temperature. Antibody-antigen complex were then detected using western lightning plus-ECL according to the manufacturer's instructions (Perkin Elmer). Analysis was carried out by Image Quant LAS-4000mini (GE imagination at work).

### Inhibition assays

BEAS-2B cells were co-cultured with Eol-1 cells in the presence of PI3 kinase inhibitor (LY294002), MEK1/2 inhibitor (MUO126), p38 kinase inhibitor (SB203580) or JNK inhibitor (SP600125) for 48 h, and then the supernatants were collected to measure TGF-β1 levels.

### Reverse transcriptase polymerase chain reaction (RT-PCR)

After co-culture of BEAS-2B with eosinophils or Eol-1 cells for 48 h as described above, eosinophils were removed. Total RNA was extracted from BEAS-2B by the guanidine isothiocyanate procedure using Trizol Reagent. RNA was reverse-transcribed using oligo-dT primers and then the DNA was amplified by PCR. The sequences of the primers are as follows: for human vimentin, forward 5′-GAGAACTTTGCCGTTGAAGC-3′ and reverse 5′- GCTTCCTGTAGGTGGCAATC-3′; for human E-cadherin forward: 5′- GTATCTTCCCCGCCCTGCCAATCC-3′ and reverse 5′- CCTGGCCGATAGAATGAGACCCTG-3′; for human GAPDH, forward 5′- GTGAAGGTCGGAGTCAACGGA-3′ and reverse 5′-GGTGAAGACGCCAGTGGACTC-3′. PCR was carried for 35 cycles (E-cadherin), 27 cycles (Vimentin), 25 cycles (GAPDH), denaturation at 94°C for 30 s, annealing at 65°C for E-cadherin and GAPDH, and 59°C for vimentin for 30 s, and elongation at 72°C for 1 min; at the end of these cycles, a further extension was carried out at 72°C for 5 min. The PCR products were separated on a 2% agarose gel containing 0.01% ethidium bromide. The RNA concentration and purity were determined by UV absorption at 260∶280 using an Ultrospec 1100 pro UV/Vis spectrophotometer (Amersham Biosciences, NJ). The amount of mRNA was normalized against the GAPDH mRNA and expressed as percentage of controls.

### Biochemical analysis

The concentration of total protein in BALF was measured using a dye-binding assay (BCATM protein assay kit; Pierce, Rockford, IL, USA) according to the manufacturer's instructions. The concentrations of cytokines in BALF and cell culture supernatant were measured using commercial immunoassay kits specific for human or mouse cytokines. The immunoassay kits for measuring interferon-γ (IFN-γ), interleukin-13 (IL-13), monocyte chemoattractant protein-1 (MCP-1), and TGF-β1were purchased from BD Biosciences Pharmingen (San Diego, CA). All cytokines were measured according to the manufacturer's instructions.

### Statistical analysis

All data were expressed as the mean ± SEM. The statistical difference between two variables was calculated by the Mann-Whitney U test, and that between three or more variables by analysis of variance with *post hoc* analysis using the Bonferroni test. Statistical analyses were performed using the StatView 4.5 package for Macintosh (Abacus Concepts, Berkeley, CA, USA). A p<0.05 was considered as significantly different.

## Supporting Information

Figure S1 **Lung distribution and time course of differential cell count after intra-tracheal instillation of eosinophils.** (A) After 3 h of intratracheal instillation, carboxyfluorescein succinimidyl ester(CFSE)-labeled bone marrow-derived eosinophils were detected in the bronchial walls and alveolar interstitial spaces (n = 3). In a separate experiment, eosinophils were instilled in the lungs and differential cell count was performed. (B) Eosinophils almost disappeared at 72 h but lymphocytes started to increase at 24 h and 72 h of intratracheal instillation. (C) Peribronchial infiltration of mononuclear cells was detected after 24 h and 72 h of eosinophil instillation. (D) The BALF concentration of TGF-β1 tended to increase at 24 h and 72 h of eosinophil instillation.
(TIF)

Figure S2 **Direct-contact and activation of Eol-1 cells are necessary for induction of EMT.** (A) BEAS-2B cells co-cultured in direct contact with EoL-1s. (B) BEAS-2B cells co-cultured with EoL-1 but using Boyden chamber. (C) TGF-β1 levels in the cell supernatant (D) BEAS-2B cells co-cultured with living EoL-1 cells. (E) BEAS-2B cells co-cultured with 2% paraformaldehyde-fixed EoL-1 cells. (F) Analysis of TGF-β1 levels in the supernatant. TGF-β1 levels were measured by ELISA. The scale bars indicate 50 µm. Data are expressed as means ± SEM. **P<0.05.
(TIF)
