# Supplementary figures and images for: Eosinophils Promote Epithelial to Mesenchymal Transition of Bronchial Epithelial Cells
Source: PLoS One. 2013 May 21;8(5):e64281. doi: 10.1371/journal.pone.0064281 (PMC3660301; doi:10.1371/journal.pone.0064281)

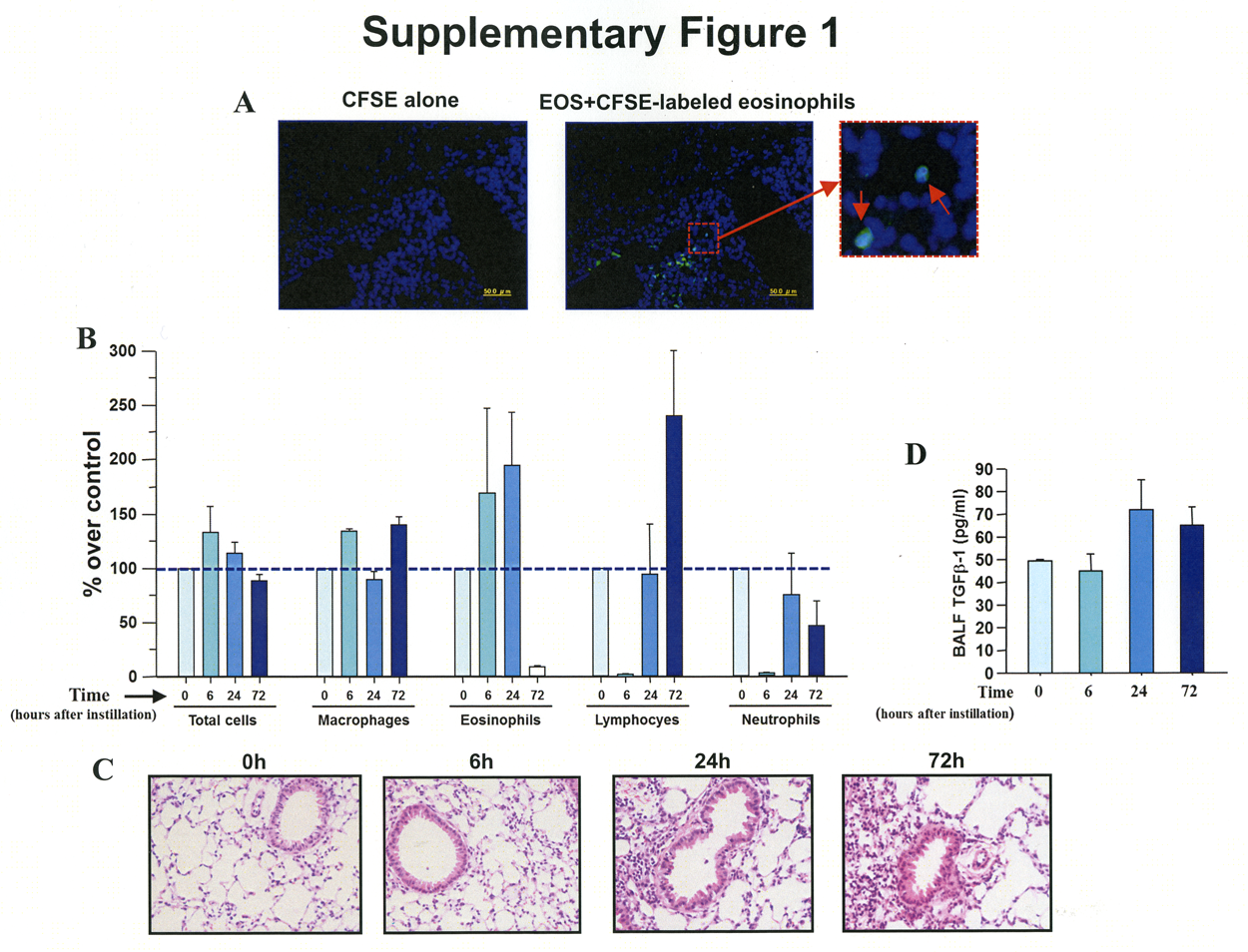

Supplement: Figure S1 — Lung distribution and time course of differential cell count after intra-tracheal instillation of eosinophils. (A) After 3 h of intratracheal instillation, carboxyfluorescein succinimidyl ester(CFSE)-labeled bone marrow-derived eosinophils were detected in the bronchial walls and alveolar interstitial spaces (n = 3). In a separate experiment, eosinophils were instilled in the lungs and differential cell count was performed. (B) Eosinophils almost disappeared at 72 h but lymphocytes started to increase at 24 h and 72 h of intratracheal instillation. (C) Peribronchial infiltration of mononuclear cells was detected after 24 h and 72 h of eosinophil instillation. (D) The BALF concentration of TGF-β1 tended to increase at 24 h and 72 h of eosinophil instillation. (TIF) [file pone.0064281.s001.tif]

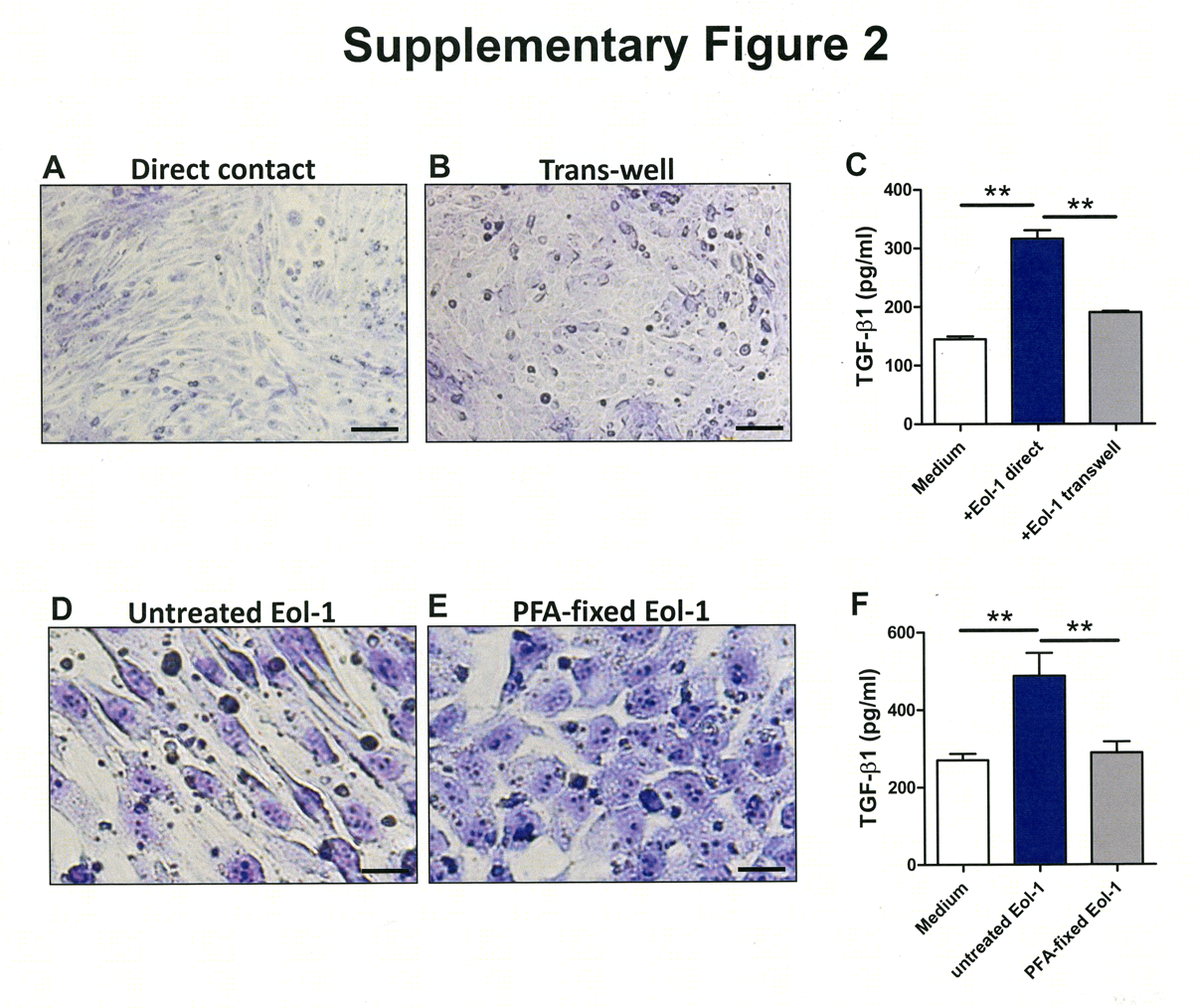

Supplement: Figure S2 — Direct-contact and activation of Eol-1 cells are necessary for induction of EMT. (A) BEAS-2B cells co-cultured in direct contact with EoL-1s. (B) BEAS-2B cells co-cultured with EoL-1 but using Boyden chamber. (C) TGF-β1 levels in the cell supernatant (D) BEAS-2B cells co-cultured with living EoL-1 cells. (E) BEAS-2B cells co-cultured with 2% paraformaldehyde-fixed EoL-1 cells. (F) Analysis of TGF-β1 levels in the supernatant. TGF-β1 levels were measured by ELISA. The scale bars indicate 50 µm. Data are expressed as means ± SEM. **P<0.05. (TIF) [file pone.0064281.s002.tif]
